# Low-dose naloxone for prophylaxis of sufentanil-induced choking and postoperative nausea and vomiting

**DOI:** 10.3389/fphar.2022.1050847

**Published:** 2022-11-18

**Authors:** Yiling Qian, Zhifei Huang, Guilong Wang, Jinghong Han, Difei Zhou, Hailei Ding, Xin Zhang

**Affiliations:** ^1^ Jiangsu Province Key Laboratory of Anesthesiology, Xuzhou Medical University, Xuzhou, Jiangsu, China; ^2^ Department of Anesthesiology, The Affiliated Wuxi People’s Hospital of Nanjing Medical University, Wuxi, Jiangsu, China; ^3^ Jiangsu Province Key Laboratory of Anesthesia and Analgesia Application Technology, Xuzhou Medical University, Xuzhou, Jiangsu, China; ^4^ Center for Translational Pain Medicine, Department of Anesthesiology, Duke University School of Medicine, Durham, NC, United States; ^5^ NMPA Key Laboratory for Research and Evaluation of Narcotic and Psychotropic Drugs, Xuzhou, Jiangsu, China

**Keywords:** sufentanil, naloxone, postoperative nausea and vomiting (PONV), sufentanil-induced cough (SIC), prophylaxis

## Abstract

Sufentanil, a potent opioid, serves as the first option for perioperative analgesia owing to its analgesic effect, long duration and stable hemodynamics, whereas its side effects frequently blunt its application. The intravenous (IV) injection of sufentanil during anesthesia induction has high incidence of choking or bucking reaction, which is defined as sufentanil-induced cough (SIC). Moreover, postoperative nausea and vomiting (PONV) is a common and stressful complication, which is also related to the usage of opioid. High incidence of PONV is reported in the patients with SIC. Hence, we sought to determine whether naloxone, an opioid antagonist, at low dose would decrease the incidences of SIC and PONV. 216 female patients undergoing gynecological laparoscopic operation (<2 h) under general anesthesia were recruited in this study, and randomly assigned into two groups: Group N (patients receiving naloxone and Group C (patients receiving vehicle). Sufentanil (0.5 μg/kg within 5 s) was given in anesthesia induction, and low-dose naloxone (1.25 μg/kg) or identical vehicle was initially injected 5 min prior to induction, with the incidence and severity of SIC estimated. Subsequently, naloxone or vehicle was continuously infused at the rate of 0.5 μg/kg/h in the initiation of operation until the end of the operation, and the transverse abdominal fascia block (TAP) was performed for postoperative analgesia. The PONV profiles such as incidence and the severity, grading, and the frequencies of antiemetic usage within 24 h were evaluated, with VAS scores and remedial measures for analgesia during the first 24 h postoperatively were recorded. Our results revealed that one bolus of low-dose naloxone prior to the induction significantly mitigated the incidence of SIC, and intraoperative continuous infusion of low-dose naloxone reduced the incidence and the severity of PONV, so that the postoperative VAS scores and further remedial analgesia were not altered. These results not only provide clinical solutions for prophylaxis of SIC and PONV, but also suggests that opioids may act as a key role in both SIC and PONV, whereas opioid antagonist may hit two tasks with one stone. Moreover, further investigations are required to address the underlying mechanism of SIC and PONV.

**Clinical Trial Registration**: [www.chictr.org.cn], identifier [ChiCTR2200064865].

## Introduction

Sufentanil is widely applied as a potent analgesic in induction and maintenance of general anesthesia due to its beneficial properties, such as its analgesic potency, long duration, and hemodynamic stability (Meijer et al., 2018; van de Donk et al., 2018). However, the adverse reactions of sufentanil during perioperative period should not be ignored (Miao et al., 2021). In the course of intravenous (IV) injection of sufentanil during anesthesia induction, cough is the most common adverse event, which is defined as sufentanil-induced cough (SIC) (Agarwal et al., 2007). Prevalence of SIC varies between 18% and 48% in un-pretreated patients. In some cases, SIC episodes are explosive or spasmodic, which would precipitate severe hemodynamic fluctuations. Even a sharp elevation of intracranial pressure or intrapulmonary pressure could be disastrous (Shen et al., 2008; He et al., 2018).

PONV is a frequent and stressful complication even in the recovery room. It usually occurs within 24 h after general anesthesia, rendering great pain, disturbances of water and electrolytes in patients, and in severe cases, dehiscence could result (Liu et al., 2015; Cao et al., 2017). The incidence of PONV is up to 70%–80% in high-risk groups, including female patients who undergo laparoscopy, prolonged operation duration and anesthetic medication, etc. PONV has been recognized to be related to the application of sufentanil (Lee et al., 2020; Massoth et al., 2021). Therefore, it is essential for an anesthesiologist to minimize sufentanil-related PONV.

Interestingly, studies have shown that both SIC and PONV are “by-products” as adverse events of sufentanil medication, with a close relationship, which was described that high intraoperative incidence of SIC was echoed by the high incidence of PONV (Ziemann-Gimmel et al., 2014). Choking reaction is a risk factor for PONV. Meanwhile, researches on the prevention and therapy of SIC or PONV depict a phenomenon of diversity and prosperity (Tramer, 2007; Lin et al., 2019; Liu et al., 2019). Since both SIC and PONV are induced by sufentanil application, there might be the identical mechanism for their occurrence. Hence, we speculated that there might be some drugs that can obviate SIC and PONV effectively and simultaneously. Naloxone, an opioid receptor antagonist, is often adopted to antagonize the residual effects of opioids post-operatively (Dunne, 2018; Sharifi et al., 2021). Recently, low-dose naloxone (roughly defined as 0.05 μg/kg∼1 μg/kg) has been reported to mitigate the opioid-induced nausea and vomiting, with unaltered analgesic effect (Barrons and Woods, 2017). Additionally, a study by Zhang et al. has revealed that combination of intravenous sufentanil and low-dose naloxone (0.25 μg kg^−1^.h^−1^) not only retains the analgesic effect of sufentanil, but also reduces the occurrence of PONV and pruritus in patients undergoing laparoscopic cholecystectomy. For preventing SIC, ketorolac, dezocine and butorphanol as well as nalmefene, a new opioid receptor antagonist, have shown their efficacy (Osborn et al., 2010; Zou et al., 2019; Tian et al., 2020; Xiong et al., 2020). To our knowledge, there is no study addressing the effectiveness of naloxone on preventing SIC. Therefore, we aim to explore the prophylactic application of low-dose naloxone and its therapeutic effect on SIC and PONV, and investigate the possible underlying mechanism.

## Materials and methods

The present study was approved by the Institutional Research Ethics Committee of the Wuxi People’s Hospital of Nanjing Medical University. The trial was registered in the Chinese Clinical Trial Registry (ChiCTR2200064865). All patients provided written informed consents. The trial was performed in accordance with the principles of the Helsinki Declaration, and adhered to CONSORT guidelines.

A total of 216 adult female patients with ASA physical status I or II were recruited in this study. The patients were scheduled for elective gynecological laparoscopic surgery under general anesthesia in the affiliated Wuxi People’s Hospital of Nanjing medical university, between January 2022 and August 2022. The exclusion criteria were as follows: history of asthma, chronic cough, and upper respiratory tract infection within 2 weeks prior to recruitment; history of peptic ulceration or bleeding, heart disease, aneurysm, hepatopathy or nephropathy; history of gastroesophageal reflux disease (GERD) and gastric retention causing nausea and vomiting. The patients who took analgesics or antiemetic agents prior to surgery were also excluded. Later, the removal criteria for the ineligible patients were as follows: referral to laparotomy, the operative duration of over 2 h, and other reasons.

Accordingly, 216 patients were randomly assigned in a 1:1 ratio into the naloxone group (Group N, *n* = 108) and the control group (Group C, *n* = 108) *via* a computer-generated randomization list, ensuring no premedication. In the operating theater, noninvasive blood pressure (NBP), pulse oxygen saturation (SpO_2_), electrocardiograms (ECGs) and bispectral index (BIS) were routinely monitored. Patients were cannulated through the median cubital vein of the forearm with a 20G venous trocar needle. Five minutes prior to general anesthesia induction, patients in Group N underwent intravenous injection of naloxone 1.25 μg/kg (diluted to 20 μg/ml by normal saline) within 3 s, while those in Group C received 5 ml of normal saline alone. The naloxone or normal saline was prepared by an anesthetic nurse and administered by an experienced anesthesiologist, both of whom were blinded to the procedures. All patients were given 100% oxygen *via* a face mask at the rate of 6 L/min for 2 min. General anesthesia was induced with a bolus of sufentanil at a dose of 0.5 μg/kg (diluted to 5 μg/ml by normal saline) administered within 5 s intravenously, and 1 min later, midazolam (0.04 mg/kg), propofol (2.5 mg/kg) and cis-atracurium (0.25 mg/kg) were sequentially infused. Endotracheal intubation was performed using a GlideScope. At the initiation of operation, additional 0.2 mg/kg of sufentanil was applied. The maintenance of general anesthesia was conducted under propofol (4–6 mg/kg/h), remifentanil (0.1–0.3 μg/kg/min) and cis-atracurium (0.2 mg/kg/h). The anesthesia depth was adjusted based on the BIS between 40 and 60. During the operation, patients in Group N were intravenously pumped with naloxone at a rate of 0.5 μg/kg/h, while the patients in Group C received the identical vehicle. At the end of the operation, all maintenance drugs were terminated and 20 ml of 0.33% ropivacaine hydrochloride was applied for TAP block bilaterally for multimodal analgesia. Thereafter, the patients were delivered to the anesthesia recovery room for tracheal extubation, and allowed for transferring to the ward when appropriate. The number of cough or choke within 1 min after sufentanil injection was recorded, and the severity was graded depending on the cough frequency (mild, 1–2; moderate, 3–4; severe, ≥ 5) (Tian et al., 2020). The mean arterial pressure (MAP), heart rate (HR) and pulse oxygen saturation (SPO_2_) were recorded at the following time-points: T0, prior to pretreatment of naloxone or normal saline, i.e., the baseline value; T1, 5 min after naloxone treatment; T2, prior to intubation; T3, 1 min after intubation; and T4, 5 min after intubation. Anesthetic dosages of sufentanil, propofol, remifentanil and cis-atracurium between two groups were also documented.

Postoperatively, the incidences of adverse reactions were recorded and evaluated, including depressed respiration, vertigo and lethargy, delayed emergence, and restlessness in the emergence period. The incidence and the severity of PONV and application of antiemetic within 24 h postoperatively also recorded. In addition, the postoperative incision pain in the patients were evaluated by the VAS scoring method, with a scale of 0–10. The association of SIC and PONV was also analyzed in patients in Group N. The primary outcome was the profile of SCI and PONV. Secondary outcomes included VAS scores and remedial measures for analgesia during the first 24 h postoperatively.

### Statistical analysis

SPSS 23.0 software (IBM Corp. Armonk, NY, United States) was performed for statistical analysis. The presented data were evaluated for normal distributions by the Kolmogorov–Smirnov test. Measurement data are expressed as the mean ± standard deviation, and Student’s t-test was employed to assess the intergroup differences. The differences in ranked data were analyzed by the Mann–Whitney U test. The chi-square test or Fisher’s exact test was adopted to assess the difference in categorical data presented as absolute or relative effect sizes. A *p*-value < 0.05 was considered significant.

## Results

**FIGURE 1:**
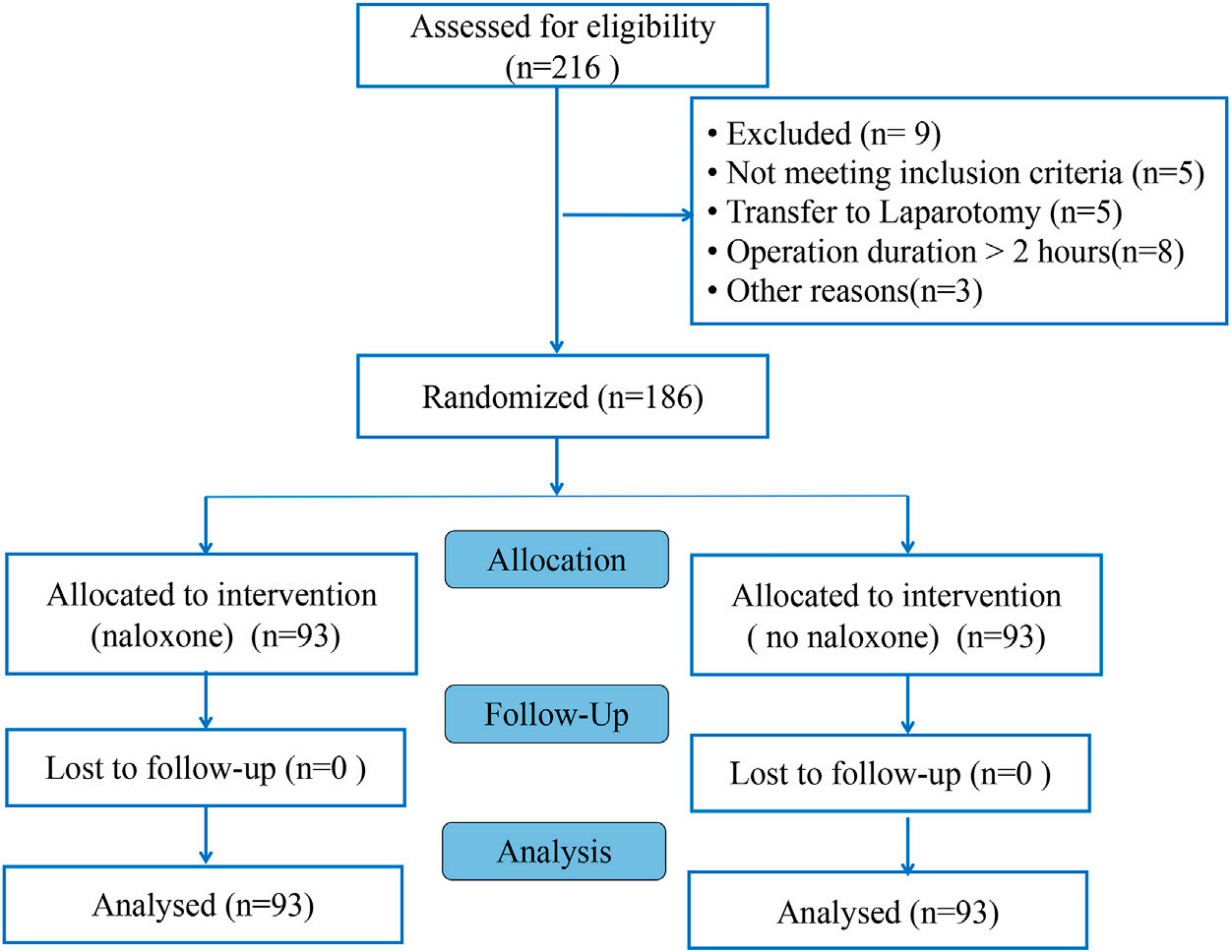
CONSORT flow diagram.

A total of 216 adult female patients with ASA physical status I or II, aged 22–68 years, weighing 44–82 kg, with BMIs between 19.7 and 28.5 kg/m^2^, were recruited in this study. Of these 216 patients recruited, 9 patients refused to participate in the study and 5 patients did not meet the inclusion criteria. 16 patients were removed from the study for referral to laparotomy (n = 5), the duration of operation of over two hours (n = 8) and other reasons (*n* = 3). Consequently, 186 eligible patients were randomly allocated into two groups: 93 cases in each group (Figure 1). Characteristics of patients, surgery and anesthesia profiles were comparable between groups (*p* > 0.05) (Table 1). There was no significant difference in the dosage of sufentanil, midazolam, propofol, remifentanil and vecuronium between the two groups (*p* > 0.05) (Table 2). No significant differences were observed in MAP, HR, SPO_2_ and BIS between the two groups at T0, T1, T2, T3 and T4. (*p* > 0.05) (Table 3). Adverse reactions did not differ significantly during the recovery period between the groups. (*p* > 0.05) (Table 4).

**TABLE 1:** Demographic characteristics of the patients between the two groups.

	Group N (93)	Group C (93)	*p*-value
Age (yr)	48.8 ± 10.4	50.2 ± 9.8	0.346
Hight (cm)	162.2 ± 16.6	165.4 ± 20.4	0.242
Weight (kg)	58.8 ± 9.2	60.4 ± 8.8	0.227
BMI(kg m–2)	23.8 ± 2.4	23.2 ± 2.1	0.071
ASA physical status (I/II)	63/30	70/23	0.251
Medical conditions (hypertension/diabetes/pulmonary disease)	20/4/2	15/5/1	0.438
Pre hisroty of PONV	3	2	0.638
Duration of surgery (min)	80.4 ± 17.8	75.3 ± 18.5	0.057
Duration of anaesthesia (min)	96.3 ± 19.4	98.2 ± 18.7	0.497

Data were expressed as the number and mean ± SD.

**TABLE 2:** Comparison of anesthetic dosages between the two groups.

	Group N (93)	Group C (93)	p-value
Midazolam (mg)	2.5 ± 0.5	2.2 ± 0.4	0.134
Sufentanil (ug)	44.8 ± 9.4	42.6 ± 10.0	0.123
Propofol (mg)	350.8 ± 45.6	336.4 ± 40.4	0.147
Cisatracurium (mg)	10.8 ± 3.2	10.4 ± 2.8	0.366
Remifentanil (ug)	465.5 ± 58.6	450.8 ± 42.1	0.072

Data were expressed as the number and mean ± SD.

**TABLE 3:** Comparison of MAP, HR, SpO_2_ and BIS values at different time points between the two groups (*n* = 186).

	Groups	T0	T1	T2	T3	T4
HR (bpm)	Group N	78.3 ± 7.2	77.5 ± 8.3	65.7 ± 7.4	74.2 ± 6.9	68.5 ± 6.6
	Group C	80.9 ± 8.1	79.2 ± 7.2	65.9 ± 6.4	79.4 ± 7.2	70.3 ± 6.8
MAP (mmHg)	Group N	88.6 ± 10.4	88.2 ± 9.6	74.5 ± 7.3	85.3 ± 10.2	80.2 ± 6.7
	Group C	85.4 ± 9.6	84.4 ± 9.2	72.2 ± 8.2	84.2 ± 8.9	82.3 ± 7.1
SpO2(%)	Group N	97.1 ±1.0	98.8 ±0.8	99.0 ± 0.3	99.0 ± 0.5	99.0 ± 0.6
	Group C	97.9 ±0.6	98.2 ±0.8	99.0 ±0.7	99.2 ± 0.5	99.2 ± 0.5
BIS	Group N	96.4 ± 1.3	92.4 ± 3.4	40.5 ± 3.8	44.8 ± 4.2	48.2 ± 5.6
	Group C	97.2 ± 1.8	91.8 ± 3.5	39.5 ± 4.2	48.2 ± 5.2	50.2 ± 5.9

Data were expressed as the number and mean ± SD.

**TABLE 4:** Comparison of adverse reactions during the recovery period between the two groups (n (%)).

Complications	Group N (93)	Group C (93)	*p*-value
Depressed respiration	2 (2.2)	2 (2.2)	---
Dizziness and Drowsiness	4 (4.3)	8 (8.6)	0.249
Delay of recovery	2 (2.2)	2 (2.2)	---
Restlessness in the recovery period	3 (3.2)	1 (1.1)	0.620

The incidence and severity of cough within 1 min after sufentanil injection in Group N was significantly decreased *versus* those in the group C (*p* < 0.05) (Table 5). The incidence and severity of PONV in the Group N were also significantly reduced as compared with Group C (*p* < 0.05). The incidence of antiemetic application in Group N was also decreased *versus* Group C (*p* < 0.05) (Table 6). There were no significant differences in VAS scores and incidence of analgesic application within 24 h postoperatively between the two groups (*p* > 0.05) (Table 7).Worthily, naloxone prevented both PONV and SIC in Group N (Table 8).

**TABLE 5:** Incidence and severity of cough between the two groups.

Groups	Incidence of SIC (n (%))	Severity of SIC (n (%))			
		None	Mild	Moderate	Severe
Group N (93)	15 (16.1)	78 (83.9)	9 (9.7)	5 (5.3)	1 (1.1)
Group C (93)	34 (36.6)	59 (63.4)	13 (14)	17 (18.3)	4 (4.3)
*p*-value	0.002	0.002	0.496	0.006	0.368

**TABLE 6:** Severity of PONV and Incidence of PONV and application of antiemetics within 24 h after operation between the two groups (n (%)).

Groups	Incidence of applicationof antiemetics (n (%))	Incidence of PONV (n (%))	Severity of PONV (n (%))			
			Ⅰ	Ⅱ	Ⅲ	Ⅳ
Group N (93)	7 (7.5)	17 (17.8)	76 (82.2)	11 (12.3)	4 (4.1)	1 (1.1)
Group C (93)	20 (21.5)	37 (39.8)	56 (60.2)	24 (25.7)	9 (9.8)	4 (4.3)
*p*-value	0.007	0.001	0.001	0.015	0.250	0.368

**TABLE 7:** Comparison of the VAS score and incidence of application of analgesics within 24 h after operation between the two groups (n (%)).

Groups	Incidence of application of analgesics (n (%))	Vas scores		
		6 h	12 h	24 h
Group N	7 (7.5)	1.8 ± 0.5	2.3 ± 0.8	1.9 ± 0.6
Group C	8 (8.6)	1.5 ± 0.5	2.5 ± 0.3	2 ± 0.7
*p*-value	0.356	0.453	0.367	0.523

**TABLE 8:** The prevention of PONV and SIC by naloxone simultaneously (n (%)).

Groups	Total (n)	Without PONV (n (%))	With PONV (n (%))	Chi square test	
				Chi square value	*p*-value
Without FIC	78	67 (85.9)	11 (14.1)	5.648	0.028
With FIC	15	9 (60)	6 (40)		

## Discussion

Opioid receptor agonists are currently among the first-option agents for perioperative analgesia, which could effectively mitigate the stress level of patients, improve the comfortability, and accelerate rehabilitation. However, its accompanying adverse reactions, such as cough reaction, respiratory depression, nausea and vomiting, etc. could adversely affect the postoperative rehabilitation and quality of life of the patients. Sufentanil, fentanyl and other opioid analgesics injected intravenously during the induction period of clinical anesthesia probably contributed to coughing reactions to varying degrees within 1 min, which might confer severe consequences for patients with hypertension, pulmonary bullae, hemangioma and intracranial hypertension.

SIC is subject to many factors, including the route of administration, concentration of medication, velocity of administration and patient conditions, etc. Studies have shown that low-dose opioids or anesthesia induction by mechanical titration of sufentanil could significantly reduce SIC (Liu et al., 2019). In addition, smoking and age are risk factors for SIC, with no significantly with gender (Oshima et al., 2006). At present, the hypotheses on the mechanism of SIC mainly includes the following (Liu et al., 2017; Shi et al., 2022): (1) Central hypothesis: afferent signals act on the cough center in the medulla oblongata and reach the effector organs (lungs, bronchi and other tissues) *via* efferent nerves, resulting in bronchoconstriction and cough; (2) Peripheral hypothesis: After activation of μ opioid receptors by opioids, afferent signals pass through stretch receptors (RARs) and presynaptic sensory C fibers located in reflex sensitive location such as the throat and carina and transform to mechanical and chemical stimulation *via* the vagus nerve, which is transmitted to the brain stem, leading to choking or bucking when transmitted through the motor efferent fibers in the vagus nerve; (3) Pharmacological action: citric acid contained in fentanyl and sufentanil is a canonical cough-causing substance. By 2020, there had been ample reports on drugs for SIC prophylaxis, such as dezocine, nalorphine, ketorolac, and magnesium sulfate, etc. (An et al., 2015; Tian et al., 2020; Wang et al., 2020; Xiong et al., 2020), but the underpinning mechanism remains obscure. PONV is another critical adverse reaction during perioperative period, with the application of opioid receptors during perioperative period aggravating the risk of PONV. The mechanism of sufentanil-induced PONV involves three aspect (Horn, 2008; Horn et al., 2014; de Boer et al., 2017): (1) Sufentanil is the excitatory mediator of chemoreceptor trigger zone (CTZ), which directly acts on CTZ to excite the vomiting center in the medulla oblongata and contributes to nausea and vomiting; (2) Sufentanil could delay gastric voiding, relax the lower esophageal sphincter, reduce gastrointestinal peristalsis, improve the sensitivity of the vestibulocochlear nerve to result in gastrointestinal discomfort; (3) Sufentanil also promotes the intestinal release of 5-hydroxytryptamine (5-HT) or excites the vagus nerve, further resulting in digestive dysfunction. At present, drugs for the prevention and treatment of PONV are aimed to block one or more receptors (Makaryus et al., 2018). Since SIC and PONV are both adverse products of sufentanil, there should be certain correlations between their pathogenesis. Accordingly, we focused on the mechanism of SIC and PONV, and revealed that both their central mechanisms are unanimously related to the medulla oblongata. We hypothesized that the occurrence of SIC and PONV might be related to the activation of the central mechanism in the medulla oblongata by the μ receptors in sufentanil, and thereafter trigger cough reaction and nausea and vomiting. Hopefully, we aimed to investigate a drug that can reverse this adverse effect to prevent and treat SIC and PONV, for which opioid receptor antagonists are candidate drugs. As a classical antagonist of μ opioid receptor, naloxone is frequently used to antagonize residual opioid drugs after general anesthesia during perioperative period (Muller-Lissner et al., 2017; Busserolles et al., 2020). At present, no reports on naloxone application to prevent SIC were available. In the therapy of PONV with naloxone, a study demonstrated that low-dose patient-controlled analgesia with epidural naloxone could effectively mitigate PONV caused by postoperative intravenous injection of sufentanil (Nimeeliya et al., 2020). Meanwhile, a dose of 5 μ g/ml naloxone could also enhance the analgesic efficacy of sufentanil (Koo et al., 2017). By competing with agonists for opioid receptors, naloxone effectively acts merely 2 min after intravenous administration, but the maintenance duration is frequently short.

In our study, we administered 1.25 μg/kg naloxone prior to the induction period of anesthesia, followed by 0.05 μg/kg sufentanil administration within 5 s during the induction period. Compared with Group C, the incidence of cough reaction in Group N was significantly reduced, coupled with the significant decrease in severity. During the operation, naloxone was continuously pumped at a rate of 0.05 μg/kg/h until the end of the operation. The incidence and severity of PONV were significantly reduced at 24th hour postoperatively in patients in Group N. Meanwhile, the incidence of other complications and postoperative VAS scores did not increase as compared with Group C. Therefore, we concluded that low-dose naloxone could indeed prevent SIC and POVN, with the novel application of the canonical drug naloxone achieving the effect of killing two birds with one stone. Based on previous studies, we speculated that the underlying mechanism by which naloxone prevents SIC and nausea and vomiting might be as follows: Opioids have a dual-action mode, which is presented as both excitability and inhibition. As for the former, opioids can be coupled with Gs protein to mediate the side effects of opioids, and as for the latter, they coupled with Gi/Go protein to mediate their analgesic effect. Low-dose naloxone might reduce the coupling of Gs protein in the medulla oblongata, thus decreasing the occurrence of nausea and vomiting. The more precise mechanism needs clarification with further animal experimentation. In addition, this study is a single-center study, which is limited by the impact of the research duration and sample size. Future researches with expanded sample size and extended research duration are invited to further address the above issues and draw more specific and comprehensive conclusions.

## Conclusion

Bolus of naloxone (1.25 μg/kg) prior to the induction significantly mitigated the incidence of SIC, and intraoperative continuous infusion of low-dose naloxone (0.05 μg/kg/h) reduced the incidence and the severity of PONV. Naloxone can prevent SCI and PONV simultaneously during the perioperative period.

## Data Availability

The original contributions presented in the study are included in the article/supplementary materials, further inquiries can be directed to the corresponding author.
